# Effective connectivity relates seizure outcome to electrode placement in responsive neurostimulation

**DOI:** 10.1093/braincomms/fcae035

**Published:** 2024-02-22

**Authors:** Katsuya Kobayashi, Kenneth N Taylor, Hossein Shahabi, Balu Krishnan, Anand Joshi, Michael J Mackow, Lauren Feldman, Omar Zamzam, Takfarinas Medani, Juan Bulacio, Andreas V Alexopoulos, Imad Najm, William Bingaman, Richard M Leahy, Dileep R Nair

**Affiliations:** Charles Shor Epilepsy Center, Cleveland Clinic Foundation, Cleveland, OH 44195, USA; Charles Shor Epilepsy Center, Cleveland Clinic Foundation, Cleveland, OH 44195, USA; Ming Hsieh Department of Electrical and Computer Engineering, University of Southern California, Los Angeles, CA 90007, USA; Charles Shor Epilepsy Center, Cleveland Clinic Foundation, Cleveland, OH 44195, USA; Ming Hsieh Department of Electrical and Computer Engineering, University of Southern California, Los Angeles, CA 90007, USA; Charles Shor Epilepsy Center, Cleveland Clinic Foundation, Cleveland, OH 44195, USA; Charles Shor Epilepsy Center, Cleveland Clinic Foundation, Cleveland, OH 44195, USA; Ming Hsieh Department of Electrical and Computer Engineering, University of Southern California, Los Angeles, CA 90007, USA; Ming Hsieh Department of Electrical and Computer Engineering, University of Southern California, Los Angeles, CA 90007, USA; Charles Shor Epilepsy Center, Cleveland Clinic Foundation, Cleveland, OH 44195, USA; Charles Shor Epilepsy Center, Cleveland Clinic Foundation, Cleveland, OH 44195, USA; Charles Shor Epilepsy Center, Cleveland Clinic Foundation, Cleveland, OH 44195, USA; Charles Shor Epilepsy Center, Cleveland Clinic Foundation, Cleveland, OH 44195, USA; Ming Hsieh Department of Electrical and Computer Engineering, University of Southern California, Los Angeles, CA 90007, USA; Charles Shor Epilepsy Center, Cleveland Clinic Foundation, Cleveland, OH 44195, USA

**Keywords:** cortico-cortical evoked potential, single-pulse electrical stimulation, responsive neurostimulation, effective connectivity, drug resistant focal epilepsy

## Abstract

Responsive neurostimulation is a closed-loop neuromodulation therapy for drug resistant focal epilepsy. Responsive neurostimulation electrodes are placed near ictal onset zones so as to enable detection of epileptiform activity and deliver electrical stimulation. There is no standard approach for determining the optimal placement of responsive neurostimulation electrodes. Clinicians make this determination based on presurgical tests, such as MRI, EEG, magnetoencephalography, ictal single-photon emission computed tomography and intracranial EEG. Currently functional connectivity measures are not being used in determining the placement of responsive neurostimulation electrodes. Cortico-cortical evoked potentials are a measure of effective functional connectivity. Cortico-cortical evoked potentials are generated by direct single-pulse electrical stimulation and can be used to investigate cortico-cortical connections *in vivo*. We hypothesized that the presence of high amplitude cortico-cortical evoked potentials, recorded during intracranial EEG monitoring, near the eventual responsive neurostimulation contact sites is predictive of better outcomes from its therapy. We retrospectively reviewed 12 patients in whom cortico-cortical evoked potentials were obtained during stereoelectroencephalography evaluation and subsequently underwent responsive neurostimulation therapy. We studied the relationship between cortico-cortical evoked potentials, the eventual responsive neurostimulation electrode locations and seizure reduction. Directional connectivity indicated by cortico-cortical evoked potentials can categorize stereoelectroencephalography electrodes as either receiver nodes/in-degree (an area of greater inward connectivity) or projection nodes/out-degree (greater outward connectivity). The follow-up period for seizure reduction ranged from 1.3–4.8 years (median 2.7) after responsive neurostimulation therapy started. Stereoelectroencephalography electrodes closest to the eventual responsive neurostimulation contact site tended to show larger in-degree cortico-cortical evoked potentials, especially for the early latency cortico-cortical evoked potentials period (10–60 ms period) in six out of 12 patients. Stereoelectroencephalography electrodes closest to the responsive neurostimulation contacts (≤5 mm) also had greater significant out-degree in the early cortico-cortical evoked potentials latency period than those further away (≥10 mm) (*P* < 0.05). Additionally, significant correlation was noted between in-degree cortico-cortical evoked potentials and greater seizure reduction with responsive neurostimulation therapy at its most effective period (*P* < 0.05). These findings suggest that functional connectivity determined by cortico-cortical evoked potentials may provide additional information that could help guide the optimal placement of responsive neurostimulation electrodes.

## Teaser

Kobayashi *et al*. report that high amplitude cortico-cortical evoked potentials near the eventual responsive neurostimulation contact sites (recorded during intracranial EEG monitoring) are predictive of better outcomes from its therapy. Effective connectivity determined by cortico-cortical evoked potentials could help guide the optimal placement of responsive neurostimulation electrodes.

## Graphical Abstract

**Graphical Abstract:**
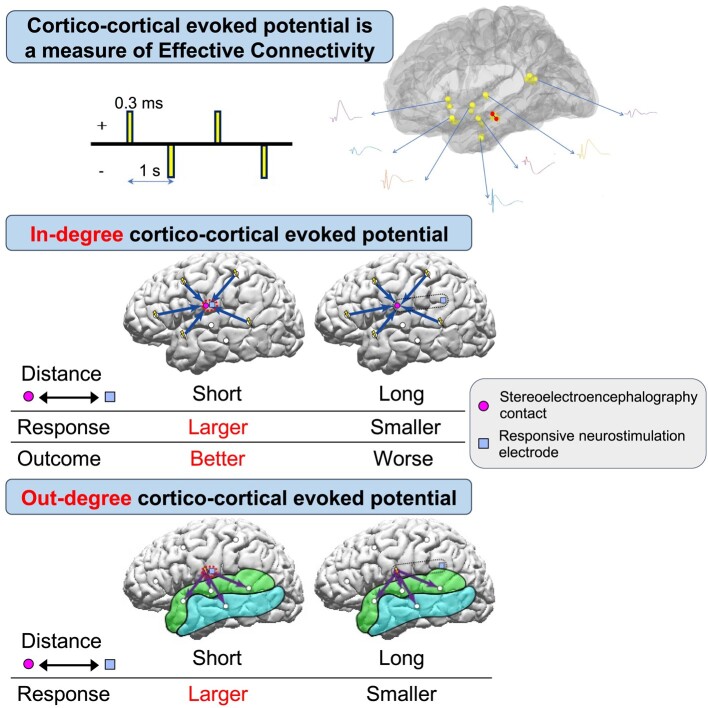

## Introduction

Approximately 30% of patients with epilepsy are drug resistant.^1^ Although epilepsy surgery is the most effective therapy for drug resistant focal epilepsy,^2^ not all are candidates for surgery. Patients with poorly localized epilepsy, multifocal epilepsy or epileptic foci that overlap with eloquent cortex are usually excluded from surgical resection.^3^ In addition, only 50–70% of patients who undergo a resection attain seizure freedom.^4-6^

Over the last two decades, neuromodulation therapies have been introduced for the treatment of epilepsy, including vagus nerve stimulation (VNS),^7,8^ responsive neurostimulation (RNS)^9,10^ and deep brain stimulation (DBS).^11,12^ RNS is a closed-loop neuromodulation therapy that delivers electrical stimuli directly to the ictal onset zone (IOZ) when epileptiform activity is detected. The RNS System has been proven safe and effective in drug resistant focal epilepsy.^9,13-17^ However, unlike VNS or DBS, RNS electrode placement is tailored to each patient’s IOZ. Placement of RNS leads can be guided by several factors including MRI, EEG, magnetoencephalography (MEG), and ictal single-photon emission computed tomography as well as intracranial EEG. RNS therapy can be directed to the IOZ, near the IOZ or target-relevant propagation networks.^18^ Functional connectivity (FC) measures are increasingly being studied to investigate epileptogenic brain networks.^19-21^ Some recent studies have introduced related strategies to identify biomarkers to prognosticate outcomes with RNS therapy, including network synchronizability (measured via intracranial EEG),^22^ functional connectivity (measured via MEG),^23^ structural connectivity (measured via tractography)^24^ and the ability of brain networks to functionally re-organize (measured via chronic intracranial EEG).^25^ Khambhati *et al*.^25^ reported that the connectivity of interictal spikes may also play a role in predicting outcome of RNS therapy, suggesting that the mechanism for RNS involves network plasticity. However, FC measures using intracranial evoked responses are not currently being used to determine RNS lead location.

Several studies have shown disruption in connectivity and changes in network topology in patients with epilepsy.^26-30^ More recently, investigators suggested that FC, based on resting state functional MRI (rs-fMRI), could be used as a tool to impact decision-making for epilepsy surgery.^31^ FC analysis, assessed by rs-fMRI pre-treatment connectivity, was shown to predict response to repetitive transcranial magnetic stimulation (rTMS) in patients with drug resistant depression.^32^ FC is defined as a statistical dependency in neurophysiologic measurements between spatially remote areas.^33^ Effective functional connectivity ascribes causal relationships between nodes in a network using models to add weighted directionality. Intracranial electrical cortical stimulation induced evoked potentials can provide a direct method to evaluate effective connectivity *in vivo* in patients with epilepsy.^34^

Recordings of local evoked potentials induced by direct electrical cortical stimulation, termed ‘direct cortical responses,’ were first performed by Adrian^35^ who found a local surface negative potential evoked by electrical stimulation of the cortical surface in various species. Single-pulse electrical stimulation (SPES) induces evoked potentials, termed cortico-cortical evoked potentials (CCEPs), which can be used to trace cortico-cortical connections *in vivo*.^36,37^ CCEPs have been extensively employed to evaluate the cortico-cortical networks associated with various normal brain functions^36-39^ and to evaluate cortical excitability and connectivity associated with areas of epileptogenicity.^40-47^ Keller *et al*.^48^ reported that both in-degree (the total number of times stimulation of any region evokes a significant CCEP at the region of interest) and out-degree (the total number of significant CCEPs observed when the region of interest is stimulated) CCEPs were higher in the seizure onset zone (SOZ) than outside SOZ, suggesting that the more epileptogenic area has larger and stronger cortico-cortical connectivity. We hypothesized that the area with larger in-degree and out-degree CCEPs could be a hub of the brain and could be a good target for RNS therapy via association fibre through which CCEPs travel. We tested this hypothesis by analysing the amplitude of intracranially recorded in-degree and out-degree CCEPs and examined its correlation with RNS outcomes. In this study, we asked the question, could CCEPs be used to optimize targets for RNS neuromodulation? In the analysis of CCEPs, the out-degree of a region represents the number of significant CCEPs elicited following stimulation of the site of interest, while the in-degree refers to the total number of significant CCEPs elicited at the site of interest upon stimulation of all other sites.^49^ These out-degree and in-degree CCEPs reflect the directional flow of information in the brain. Since seizures propagate through cortico-cortical networks, CCEPs is a unique approach to study directional connectivity of epileptogenic networks. As in-degree CCEPs of a given cortical site reflect an area of hypersynchrony, and out-degree CCEPs can be a measure of ictal propagation, we were interested in analysing both measures for the optimal placement of RNS electrodes. We reviewed the CCEPs performed in patients during stereoelectroencephalography (SEEG) evaluation who later went on to have RNS therapy. We investigated the correlations between CCEPs during SEEG and prior to the RNS placement, the eventual RNS contact location and outcomes from RNS therapy. In this study, we retrospectively investigated the degree of connectivity, as measured by CCEPs, at the point of the RNS contact placement in two scenarios: (i) the distances from the SEEG recording sites of CCEPs to the eventual closest RNS contacts for in-degree CCEPs; and (ii) distance from the SEEG stimulus sites for CCEPs to the eventual closest RNS contacts for out-degree CCEPs. Our primary hypothesis was that there exists a greater degree of both in-degree and out-degree connectivity at the point of RNS contact in patients who benefit from RNS therapy. Both in-degree and out-degree CCEPs have been reported higher in the SOZ than outside SOZ, suggesting that the more epileptogenic area has larger and stronger cortico-cortical connectivity.^48^ In-degree CCEPs may reflect the degree of hypersynchronous brain activity. Those brain regions associated with high in-degree CCEPs could be influenced by neuronal or electrical activity from distant sources. This could be one explanation for why neuromodulation therapy with a wide variety of stimulation targets can still modulate the epileptic network. In addition, we have reported that by stimulating the ictal onset zone, significant out-degree CCEPs were observed within the epileptic network.^43^ Such a finding could support the notion that FC should be considered during RNS contact placement with the goal of modulating the epileptic network as a whole.

## Materials and methods

### Patients

**Figure 1:**
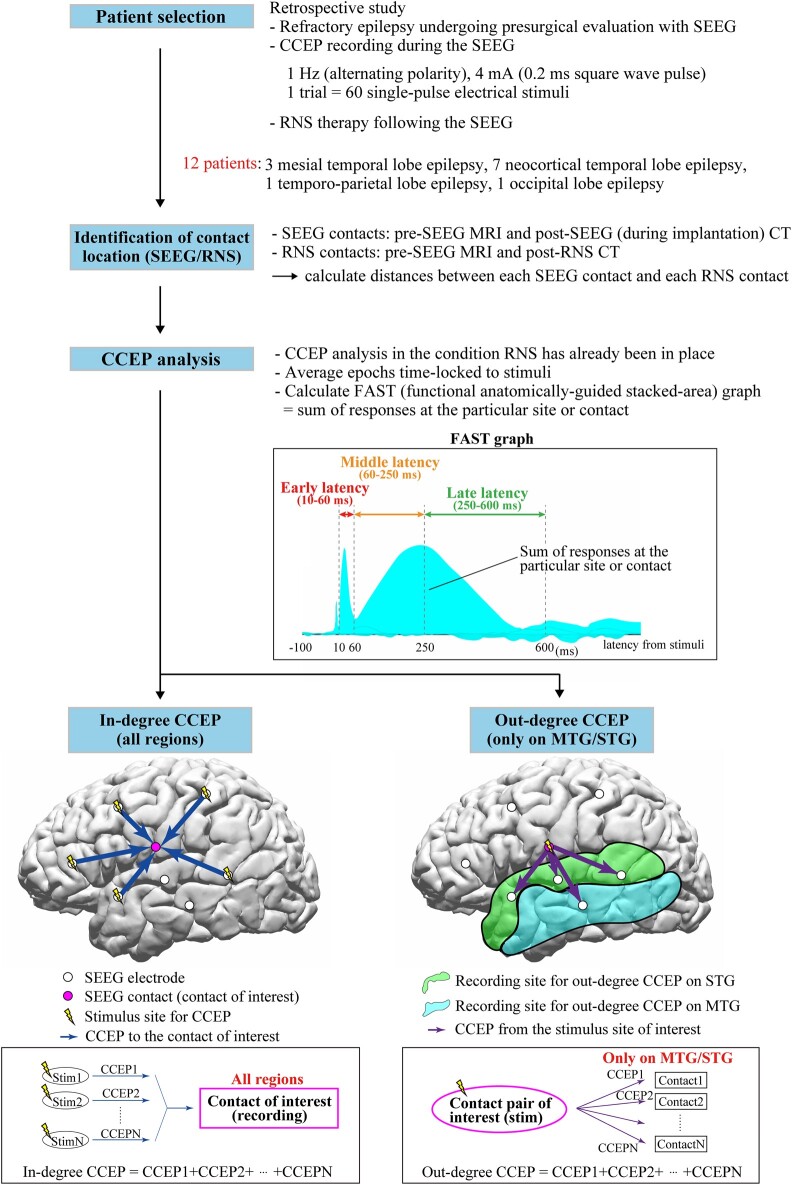
**Flowchart of whole steps of recording and analyses.** The functional anatomically-guided stacked-area (FAST) graph displays the sum of the responses at the particular area or SEEG contact to visualize and investigate ‘in-degree’ and ‘out-degree’ CCEPs. The ‘in-degree’ CCEPs indicate the sum of the potentials on the region of interest evoked by stimulating any pairs of SEEG contacts, namely, the degree of the input to the recording site. The ‘out-degree’ CCEPs indicate the sum of potentials from the region of interest, namely, the degree of output from the stimulus site. In this study, for the standardization of the CCEPs across patients, we focused on the ‘out-degree’ CCEPs from each stimulus site on only middle temporal gyrus (MTG) and superior temporal gyrus (STG), which were the only two common anatomical locations across the 12 patients. CCEPs, cortico-cortical evoked potential; SEEG, stereoelectroencephalography; RNS, responsive neurostimulation; FAST graph, functional anatomically-guided stacked-area graph; MTG, middle temporal gyrus; STG, superior temporal gyrus.

We studied 12 patients (six females) with drug resistant focal epilepsy who underwent CCEPs recordings during their presurgical SEEG evaluation and subsequently underwent RNS therapy (NeuroPace, Inc., Mountain View, CA). The median age was 26 years (range: 18–60) at time of RNS implantation, median age of epilepsy onset was 13 years (range: 2–34) (Supplementary Table 1). Prior to their SEEG evaluation, patients underwent non-invasive testing including: scalp video-EEG, MEG, brain MRI, ^18^F-fluorodeoxyglucose positron emission tomography (PET), ictal single-photon emission computerized tomography (SPECT) and neuropsychological examinations. All patients were discussed in the comprehensive patient management conferences at our centre before and after their SEEG evaluation that resulted in a decision to offer RNS therapy. Discussion at our post-SEEG patient management conference included suggestions as to where the RNS electrodes should be placed for each patient and was based solely on the ictal patterns recorded during SEEG. The planning of the location of the RNS lead was informed by IOZ SEEG location. The implanted RNS System includes a programmable neurostimulator connected to 1–4 depth electrodes or subdural strip leads with each lead containing four electrodes. Figure 1 shows a flowchart of the steps involved in the analysis. The use of RNS depth electrodes versus subdural strips was assessed individually based on location of the IOZ during patient management conference. This retrospective study protocol was approved by the Cleveland Clinic institutional review board.

### CCEPs recording during the SEEG evaluation

**Figure 2:**
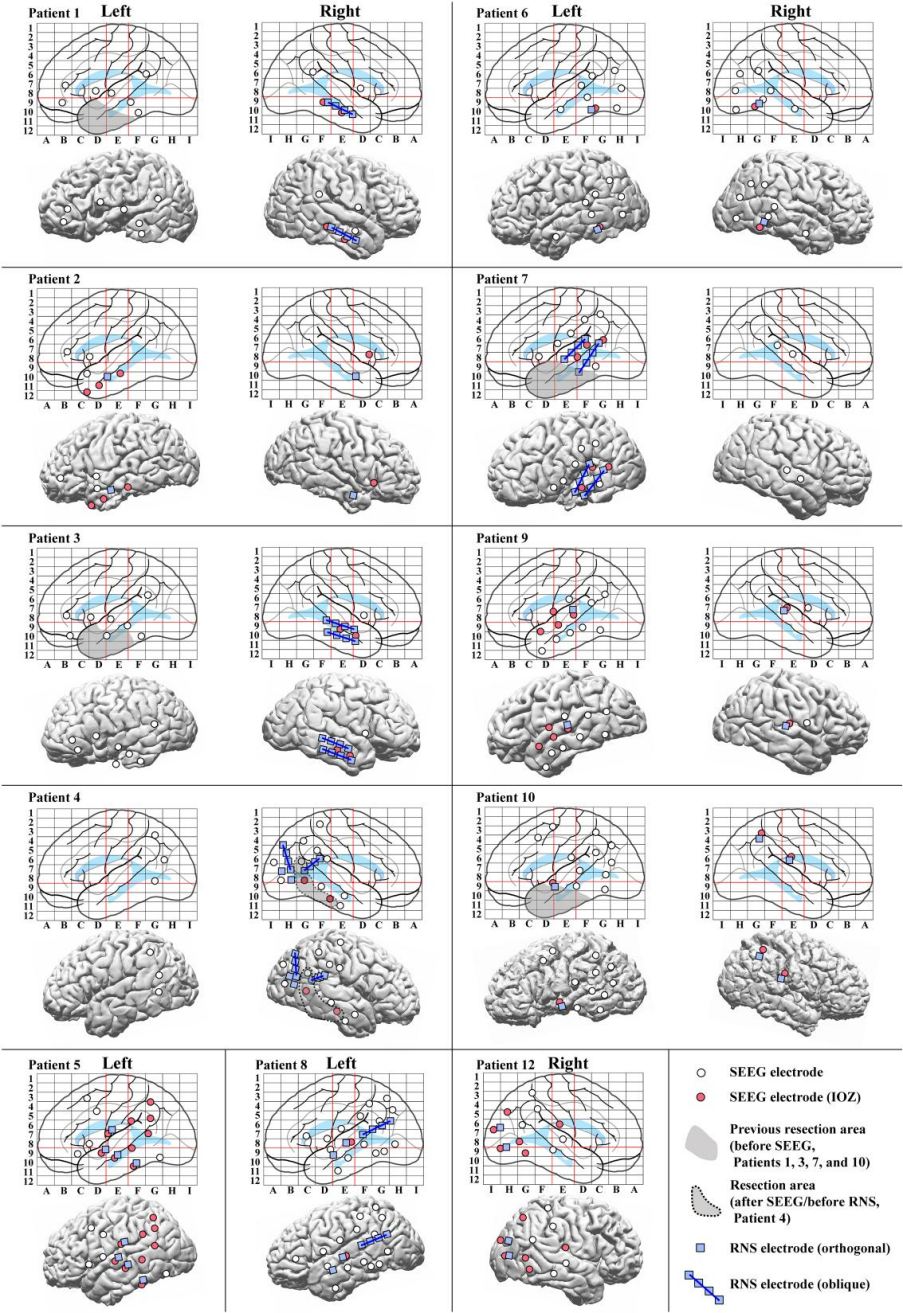
**Configurations of SEEG and RNS electrodes.** Configurations of SEEG and RNS electrodes are shown in anatomical labels in Talairach space (*top* for each patient) and on a presurgical 3D-MRI (*bottom* for each patient), except that for Patient 11 whose is shown in Fig. 3. IOZs were defined by the experts’ review of ictal SEEG. The red lines on the electrode maps indicate the vertical line passing the anterior commissure (VAC line, between **D** and **E**), the vertical line passing the posterior commissure (VPC line, between **E** and **F**) and the AC–PC line (a horizontal line between 8 and 9) in Talairach space to visualize and standardize the electrode locations of SEEG. Abbreviations: the conventions are same as for Fig. 1.

Among the 12 patients, nine underwent bilateral and three unilateral SEEG electrode implantation (two left hemisphere, one right hemisphere). The median number of implanted SEEG electrodes was 13 (range: 7–16). The median number of pairs of stimulus sites for CCEPs was 11.5 (range 3–45) and the median number of SEEG contacts implanted used for CCEPs recording was 150 (range: 65–185). The median number of contacts on middle temporal gyrus (MTG) for out-degree CCEPs analysis was 13.5 (range: 2–20) and that on superior temporal gyrus (STG) was 11 (range: 2–26). The configurations of SEEG electrodes and RNS electrodes are shown in Talairach space and on a presurgical 3D-MRI in Fig. 2. The 12 patients were classified as follows: three mesial temporal lobe epilepsy (mesial TLE), seven neocortical TLE, one temporo-parietal lobe epilepsy (T-PLE) and one occipital lobe epilepsy (OLE) according to the results of SEEG evaluation (Supplementary Table 1). Only one patient (Patient 4) underwent a resective epilepsy surgery of the right posterior basal temporal region before RNS therapy.

Depth electrodes for SEEG evaluation were made of platinum (AdTech, Integra or PMT) and implanted using the Talairach stereotactic method based on the results of presurgical non-invasive evaluation in each patient.^50^

The methodology of CCEPs evaluation has been previously reported.^36,51^ In brief, CCEPs were recorded towards the end of the SEEG evaluation when patients are restarted on their antiseizure medications. Using a constant-current stimulation device (Grass S88, Astro-Med, Inc., RI, USA), SPES at 4 mA consisting of 0.2 ms square wave pulses was repetitively applied at 1 Hz with alternating polarity through a pair of adjacent SEEG contacts in the cortices. Trials of 60 SPESs were delivered for each stimulus site (two trials of 30 SPESs). Reponses were recorded at a sampling rate of 1000 Hz (EEG-1200, Nihon Kohden, Tokyo, Japan) and bandpass filtered at 0.08–300 Hz. The reference electrode was placed on the skin at the vertex region.

### Identification of locations of RNS contacts and the correlation with SEEG contacts

The locations of the contacts relative to brain anatomy were determined by co-registering the preoperative MRI of the brain with the head CT scan for SEEG electrode locations and CT scan for RNS electrode locations. We have recently developed a semi-automated identification process of anatomical labelling for intracranial electrode contacts, details of which are described in Tayler *et al*.^52^ In brief, the preoperative MRI of each patient was imported to BrainSuite^53^ and an anatomical segmentation was performed based on the USCBrain atlas.^54^ The SEEG contacts locations are determined from the CT using the Curry software (Compumedics, NeuroScan Laboratories, Charlotte, NC, USA), and the outputs are combined to localize and automatically assign anatomical labels to each contact. The contact locations of RNS were identified in the same manner. The distances between each SEEG and each RNS contact were calculated using an in-house MATLAB script (the Mathworks, Inc., Natick, MA, USA). BrainSuite^53^ and BrainStorm software^55^ were used to confirm the locations of the contacts of SEEG and RNS electrodes within the brain volumes.

### Directional connectivity analysis using CCEPs

The process of CCEPs analysis was performed with BrainStorm using the method described by our group.^56^ After importing the raw SEEG data, excluding epochs contaminated with artefacts, we obtained CCEPs by averaging the remaining epochs time-locked to the stimulation. Baseline was taken from 100 to 5 ms before stimulation. We categorized the CCEPs stimulation site and response site by their anatomical location. The anatomical location of CCEPs performed during presurgical SEEG evaluation was retrospectively compared to the ensuing locations of the RNS contacts. We analysed whether SEEG recording and stimulus sites of CCEPs close to the ensuing RNS contacts were associated with large in-degree [the sum of the potentials in the region of interest evoked by stimulating all other pairs of SEEG contacts (Fig. 1 bottom, left)] and out-degree CCEPs [the sum of potentials from the region of interest, namely, the degree of the output from the stimulus site (Fig. 1 bottom, right)] in patients with good RNS outcomes.

To evaluate directional connectivity, we analysed in-degree and out-degree CCEPs.^48,57^ For in-degree CCEPs, we investigated the CCEPs responses seen in the SEEG contact closest to the ensuing RNS contact. The in-degree CCEPs are the sum of the potentials in the region of interest evoked by stimulating all other pairs of SEEG contacts (Fig. 1 bottom, left). For in-degree CCEPs, the analysis included responses in all regions of cortices stimulated. For out-degree CCEPs, we analysed the CCEPs responses in just two regions that were common to all patients, MTG and STG, following stimulation of site closest to the ensuing RNS contact. The out-degree CCEPs are the sum of potentials from the region of interest, namely, the degree of the output from the stimulus site (Fig. 1 bottom, right).

### Functional anatomically-guided stacked-area graph (FAST graph)

**Figure 3:**
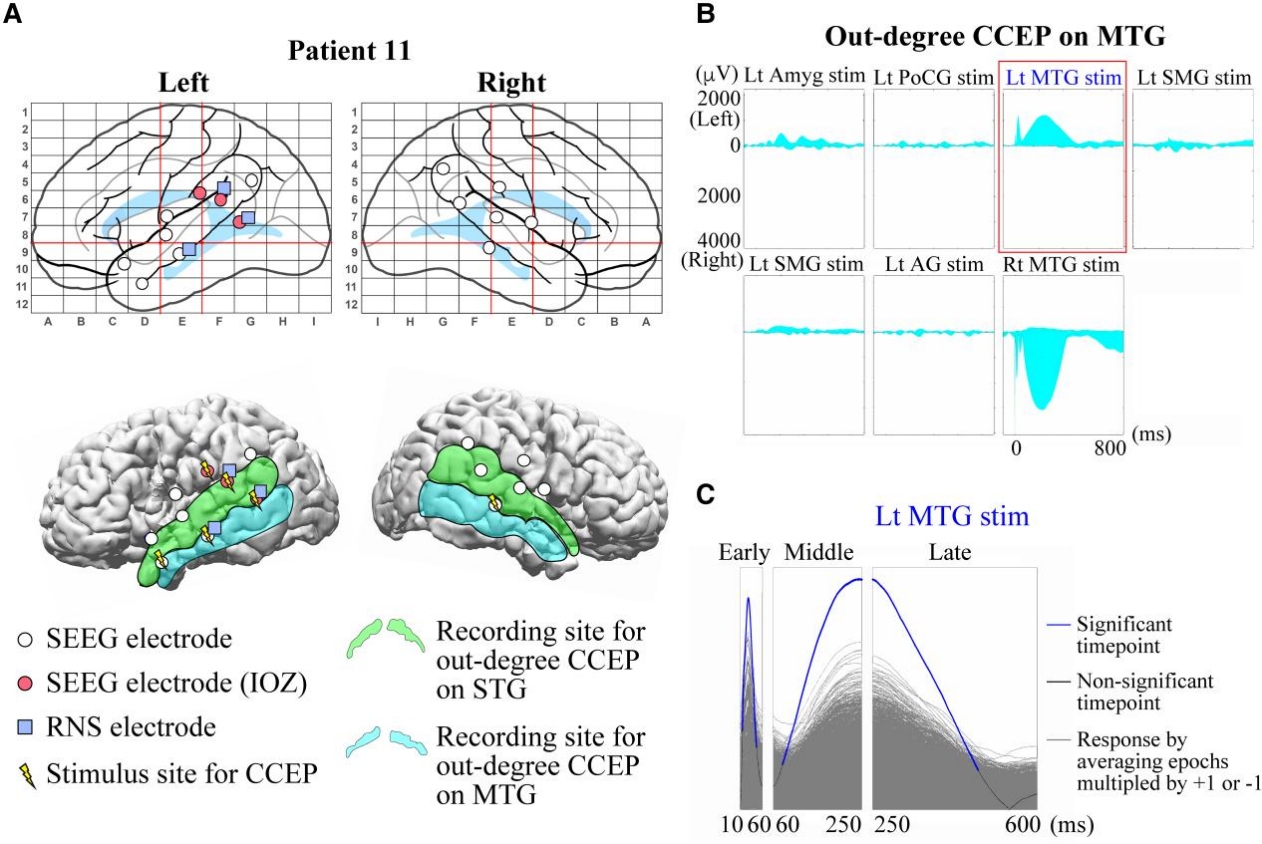
**Example of CCEPs FAST graph and a sign permutation test.** (**A**) Configurations of SEEG and RNS electrodes in a representative patient (Patient 11). (*Top*) SEEG electrodes (white circle), those judged as ictal onset zone (IOZ) (red/solid circle) and RNS electrodes (blue/filled square) are shown in anatomical labels in Talairach space. Two red vertical lines and one red horizontal line indicate VAC, VPC and AC–PC lines, respectively, as in Fig. 2. (*Bottom*) The SEEG and RNS electrodes and stimulus sites (yellow/electric mark) are displayed on a presurgical (before SEEG evaluation) 3D-MRI. The light green (STG) and the light blue (MTG) areas represent the recording sites for out-degree CCEPs. (**B**) An example of CCEPs FAST graph recorded from the SEEG contacts on MTG. (*Top*) A total of seven pairs of SEEG contacts were stimulated for the out-degree CCEPs recording in this patient. Each graph indicates the stacked area plots of the rectified out-degree CCEPs waveforms presenting the sum of the amplitudes on the left and right MTGs. The horizontal axis indicates the analysed time window (−100 to 900 ms from the stimulation). The area above 0 and below 0 suggests the sum in the left MTG and that in the right MTG, respectively. (**C**) An example of a sign permutation test. The FAST graph of the sum of the out-degree CCEPs on MTG by stimulation of a pair of SEEG contacts on Lt MTG (highlighted with a rectangle in red in Fig. 2B) was evaluated statistically for each time point. The amplitude in each epoch was randomly multiplied by either +1 or −1 and then the epochs were averaged. This procedure was repeated 5000 times for each response (grey waveforms), and they were compared with the original CCEPs waveform (blue and black waveform) to show the data-driven distribution for the statistical analysis. For the multiple comparisons, we used a false discovery rate (FDR, *α* = 0.05). The blue parts indicate the significant time points and the black parts the time points without significance. IOZ, ictal onset zone; Lt, left; Rt, right; Amyg, amygdala; PoCG, postcentral gyrus; SMG, supramarginal gyrus; AG, angular gyrus. Other conventions are same as for Figs 1 and 2.

In performing our CCEPs analysis, we generated functional anatomically-guided stacked-area (FAST) graphs of CCEPs responses. Details of the FAST graph have been previously reported by our group.^56^ In brief, area plots of rectified CCEPs waveforms are stacked to present the sum of responses, either across all contacts for a single stimulation pair (out-degree), or at a single contact across multiple stimulations (in-degree). An example of the FAST graph in a representative patient (Patient 11) is shown in Fig. 3. The locations of SEEG and RNS electrodes and stimulus sites for CCEPs in this patient are displayed in Fig. 3A. In previous studies recorded with subdural electrodes, CCEPs were typically composed of two negative potentials (early N1 and late N2), and the latency of N1 and N2 usually ranged from 10–50 ms and 100–200 ms, respectively.^36^ However, the CCEPs in patients with SEEG demonstrate more complex waveforms, likely due to variable contact locations within the six layers of cerebral cortex and white matter.^58^ In order to overcome this complexity, we used the sum of the absolute value of the CCEPs response, separated into early (10–60 ms), middle (60–250 ms) and late latency periods (250–600 ms) (Figs 1 and 3B).

For in-degree CCEPs, we calculated the CCEPs FAST graph at each SEEG contact from all stimulation sites. The responses for each SEEG contact were sorted in descending RMS order and ranked for the early, middle and late latency periods in each patient. SEEG contacts in both grey and white matters were included in the in-degree CCEPs. In our experience, CCEPs performed during SEEG can show meaningful responses in electrodes within both white and grey matters. To specify the responses of each location including white matter, we adopted a bipolar montage for the analyses of CCEPs and corresponding FAST graphs in the localization of in-degree CCEPs.^59^ For out-degree CCEPs, we grouped similar stimulation and responses in the only two common anatomical locations across patients (MTG and STG). The out-degree CCEPs FAST graphs from MTG/STG were recorded using a referential montage (referenced the scalp vertex region), since the possibility of far field responses was eliminated by sampling only the contacts in this cortical region of interest in all patients. In this study, we confirmed the area showing hyperperfusion in ictal SPECT based on our previous report demonstrating strong connectivity between the ictal onset zone and hyperperfused regions in ictal SPECT.^43^ Eleven out of 12 patients underwent an ictal SPECT examination during preoperative evaluation. The rate of significant out-degree CCEPs between patients with and without ictal hyperperfusion on MTG/STG in subtraction ictal SPECT co-registered with MRI (SISCOM) was compared.

### Outcome of RNS therapy

Seizure outcomes in patients undergoing epilepsy surgery are typically reported using the Engel classification^60^ or International League Against Epilepsy (ILAE) classification.^61^ For RNS seizure outcomes, the response to neuromodulation changes over time. Therefore, the percentage seizure reduction at one time point to the baseline seizure frequency pre-RNS therapy is more clinically meaningful.^9,16^ We applied a modified percentage outcome scale based on the seizure reduction from pre-RNS seizure frequency. These seizure outcome categories consisted of: worsened (scale −1), no change (scale 0), 1–24% seizure reduction (scale 1), 25–49% seizure reduction (scale 2), 50–74% seizure reduction (scale 3), 75–99% seizure reduction (scale 4) and 100% seizure reduction (scale 5). The follow-up period for evaluation ranged from 1.3–4.8 years (median 2.7) after RNS therapy was initiated. In two patients (#3 and #10), we could not accurately determine the seizure outcome due to the patient’s inability to report their seizure frequency reliably. Of the two patients (75–99% seizure reduction; scale 4), one had antiseizure medication (ASM) reduced and one had no change; one patient (50–74% seizure reduction; scale 3) had no ASM change; four patients (25–49% seizure reduction; scale 2), three had no change and one had ASM increased; three patients (1–24% seizure reduction; scale 1), two had no change and one had ASM increased.

### Grouping of SEEG contacts according to the distance to the ensuing closest RNS contact

We classified the SEEG contacts (for stimulation and recording in CCEPs) into four groups based on distance to the closest ensuing RNS contact: Group 1 (G1: 0–5 mm), Group 2 (G2: 5–10 mm), Group 3 (G3: 10–20 mm) and Group 4 (G4: >20 mm). Due to the number of stimulation pairs being smaller than those for the recording, we combined G3 and G4 as G3-4 (>10 mm from the closest RNS contact) for the analyses of out-degree CCEPs.

### Statistical analysis

For each latency period (early, middle and late), we compared the ranks according to the highest amplitude over the time window of in-degree CCEPs in each group described above using a Mann–Whitney U test [a *P*-value of <0.016 was considered significant for multiple comparisons (G1 versus G2, G1 versus G3 and G1 versus G4) by Bonferroni correction]. We standardized the CCEPs per patient in this way because the distribution and amplitude of CCEPs are different for each patient. We then calculated the in-degree CCEPs ratios for G1, G2 and G3 relative to G4 by using the highest amplitude of the sum of CCEPs. The mean of the highest amplitude of the sum of CCEPs in G1, G2 and G3 was divided by that in G4 for the early, middle and late latency periods in each patient. The correlations between the in-degree CCEPs ratios (G1/G4, G2/G4 and G3/G4 ratios) and the outcome of RNS therapy were evaluated by the Pearson correlation coefficient (a *P*-value of <0.016 was considered significant for each for multiple comparisons by Bonferroni correction). We examined the seizure reduction rate every visit. Variations of the outcome of RNS therapy are known to occur based on programming changes to both detection and stimulation parameters.^14^ Thus, we adopted the score at the time of the greatest seizure reduction after RNS therapy for each patient in this study.

When investigating the out-degree CCEPs, we performed a sign permutation test to extract statistically significant CCEPs for each stimulus site in the aforementioned FAST graph based on previously established methods.^62^ The details of the sign permutation test are further described by our group.^56^ In order to evaluate significance, the amplitude in each epoch was randomly multiplied by either +1 or −1 and the epochs were averaged. This procedure was repeated 5000 times for each response to generate a data-driven null distribution. This was then compared with the original CCEPs waveform for statistical analysis (Fig. 3C). For multiple comparison correction, we used a false discovery rate correction protocol (FDR, *α* = 0.05). CCEPs were deemed significant when at least 1 time point showed significance in the sign permutation test. The rate of significant out-degree CCEPs, namely the number of stimulus pairs producing significant CCEPs divided by the number of all stimulus pairs, was then calculated for MTG and STG in each patient. For group comparisons of the rate of significant out-degree CCEPs between patients with and without ictal hyperperfusion on MTG/STG in SISCOM, we applied a Mann–Whitney U test [a *P*-value of <0.016 was considered significant for multiple comparisons (early, middle and late latency periods on MTG/STG) by Bonferroni correction]. A Wilcoxon Signed-rank test was performed for the comparison between G1 and G3-4 for out-degree CCEPs (a *P*-value of <0.05 was considered significant).

An additional analysis was done for out-degree CCEPs and is available in Supplementary material.

## Results

### In-degree CCEPs to the closest RNS contacts

**Figure 4:**
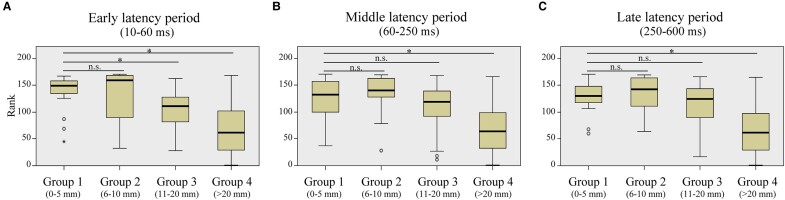
**Correlation of the in-degree CCEPs with the distance between recording site of CCEPs and the closest RNS contact (in a representative patient).** Based on the distances to the closest RNS contacts, we classified the recording sites into the four groups: Group 1 (G1: 0–5 mm), Group 2 (G2: 5–10 mm), Group 3 (G3: 10–20 mm) and Group 4 (G4: >20 mm). Each recording site was sorted by the rank of the in-degree CCEP. In this patient (Patient 11), there were significant differences between G1 and G4 for early (**A**), middle (**B**) and late (**C**) latency periods (*P* < 0.016, for all), suggesting significantly larger in-degree CCEPs in G1 compared with those in G4. There was also a significant difference between G1 and G3 for early latency period (*P* < 0.016, for both). *In this figure indicates a significant difference (*P* < 0.016), using a Mann–Whitney U test. The numbers of plots for each group are 19 for G1, 9 for G2, 33 for G3 and 110 for G4.

Following SEEG evaluation, the electrodes for RNS therapy were implanted with up to four electrodes of which only two can be connected: one electrode in one patient, two electrodes in six patients (bilaterally in three patients), three electrodes in three patients (bilaterally in one patient) and four electrodes in one patient. Figure 4 represents the ranks of all contacts sorted by the values of in-degree CCEPs in each group relative to their rank among all contacts in a representative patient (Patient 11). The rank of G1 (0–5 mm from the closest RNS contact) was significantly higher than that of G4 (>20 mm from the closest RNS contact) for the early, middle and late latency periods (*P* < 0.001, for all) and that of G3 (11–20 mm from the closest RNS contact) for the early latency period (*P* = 0.001), whereas there were no differences between G1 and G2 or between G1 and G3 (for the middle and late latency periods) (*P* > 0.016). The in-degree CCEPs were significantly higher in G1 than G4 in six patients for the early and middle latency periods (*P* < 0.016, for all), and in five patients for the late latency periods (*P* < 0.016, for all). The in-degree CCEPs were also significantly higher in G1 than G3 in four patients for the middle and late latency periods (*P* < 0.016, for all) and in five patients for the early latency period (*P* < 0.016, for all). There was no difference between G1 and G2 for all the latency periods (early, middle and late) in any of the patients (*P* > 0.016). This analysis could not be assessed in two out of 12 patients due to lack of CCEPs data in proximity to the eventual RNS contact. The results of the statistical tests for all patients are summarized in Table 1.

**Table 1:** Comparison of in-degree CCEPs between Group 1 (0–5 mm from RNS contacts) and Groups 2–4

Patient	Group 1 (0–5 mm) versus Group 2 (5–10 mm)			Group 1 (0–5 mm) versus Group 3 (10–20 mm)			Group 1 (0–5 mm) versus Group 4 (>20 mm)		
	Early	Middle	Late	Early	Middle	Late	Early	Middle	Late
1	0.021	0.040	0.029	0.006*	0.015*	0.015*	<0.001*	<0.001*	<0.001*
2	n.a.	n.a.	n.a.	n.a.	n.a.	n.a.	n.a.	n.a.	n.a.
3	0.400	0.200	0.400	0.643	0.143	0.143	0.148	0.895	0.876
4	n.a.	n.a.	n.a.	n.a.	n.a.	n.a.	n.a.	n.a.	n.a.
5	0.128	0.248	0.248	<0.001*	<0.001*	0.001*	0.001*	0.005*	0.015*
6	0.868	0.616	0.525	<0.001*	<0.001*	<0.001*	<0.001*	<0.001*	<0.001*
7	0.029	0.396	0.672	0.051	0.786	0.197	0.001*	0.014*	0.439
8	0.042	0.148	0.057	<0.001*	0.058	0.008*	<0.001*	<0.001*	<0.001*
9	0.842	1.000	1.000	0.482	0.750	0.841	0.619	0.431	0.436
10	0.081	0.938	0.815	0.118	0.016*	0.104	0.548	0.042	0.113
11	0.357	0.699	0.809	0.001*	0.180	0.213	<0.001*	<0.001*	<0.001*
12	0.404	0.525	0.404	0.274	0.904	0.659	0.195	0.224	0.576

n.a., not available. A *P*-value of <0.016 (*) was considered significant for all the statistical tests in the comparison between the Group 1 and Groups 2–4 for multiple comparison with Bonferroni correction.

### Correlation between in-degree CCEPs ratio and RNS outcomes

**Figure 5:**
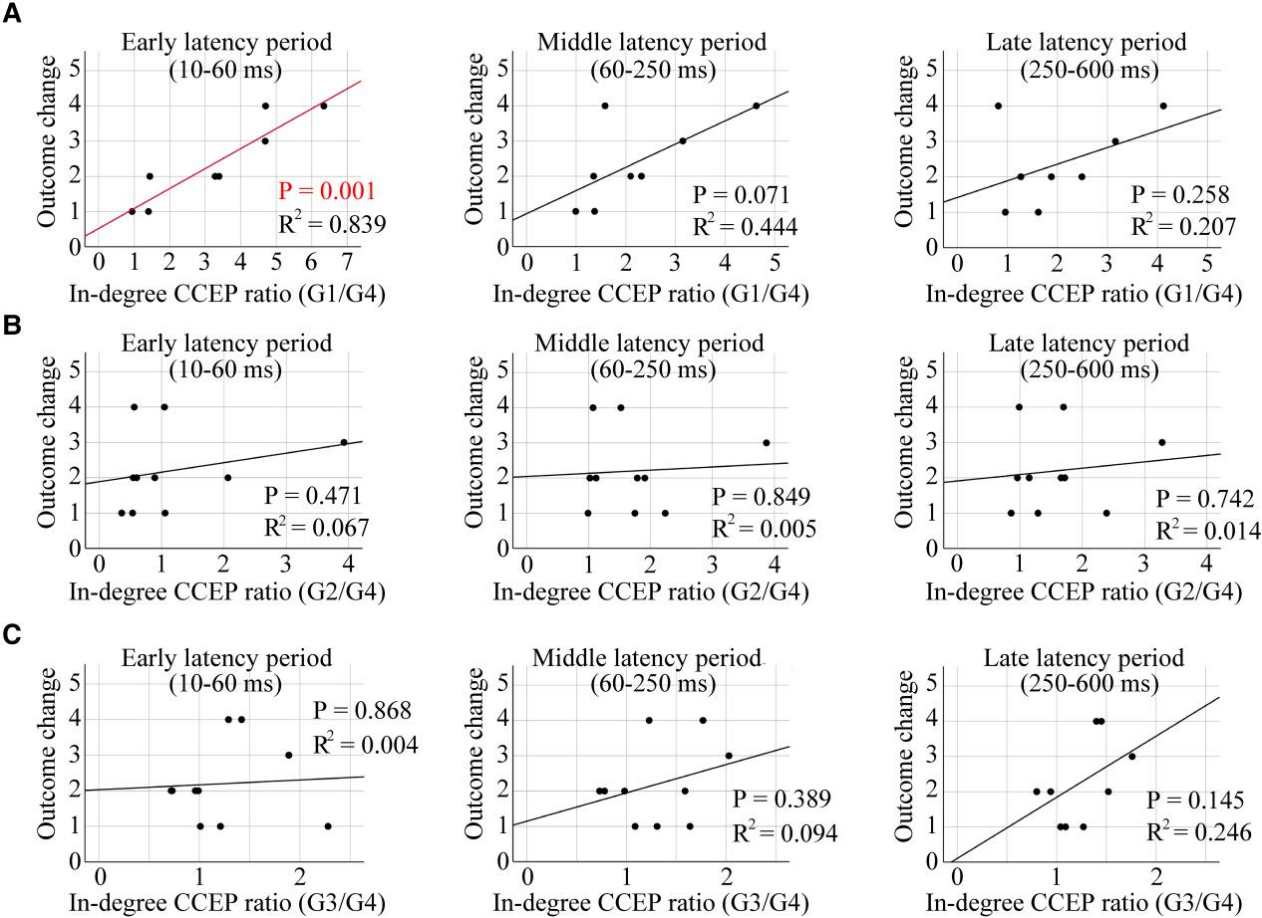
**Correlation of the in-degree CCEPs ratio with the outcome value of RNS therapy.** A significant correlation was seen between the in-degree CCEPs ratios of G1/G4 and the outcome for the early latency period (*P* = 0.001, *R*^2^ = 0.839), while there were no correlations for middle and late latency periods (*P* > 0.05, for both) (**A**). Individual data points on these plots for panel **A** are shown in Supplementary Table 2. There were no correlations between the in-degree CCEPs ratios of G2/G4 or G3/G4 and the outcome (early, middle and late latency periods) (*P* > 0.05, for all) (**B** and **C**). The Pearson correlation coefficient was used for all the statistical tests.

Figure 5 shows the correlations between the in-degree CCEPs ratios (for early, middle and late latency periods) and RNS seizure outcomes. There was a significant correlation between the early latency period of the in-degree CCEPs ratios of G1/G4 and outcome (*P* = 0.001, *R*^2^ = 0.839). This result implies that high in-degree CCEPs early latency period ratios correlate with better outcome of RNS therapy, whereas there were no significant correlations for middle and late latency periods (*P* > 0.05) (Fig. 5A). No significant correlations were seen between the in-degree CCEPs ratios of G2/G4 or G3/G4 and the outcome for early, middle and late latency periods (*P* > 0.05, for all) (Fig 5B and C).

### Ratio of stimulus sites producing significant out-degree CCEPs on MTG/STG

In all patients, FAST graphs for the out-degree CCEPs on MTG/STG were constructed and a sign permutation test was performed. The ratios of stimulus sites with significant out-degree CCEPs on MTG and STG to all stimulus sites for early, middle and late latency periods were calculated in each patient. There was no association of significant out-degree CCEPs (either early, middle or late latency period) to the epilepsy classification. Five patients (Patients 3, 5, 6, 7, 10) showed hyperperfusion on MTG/STG in SISCOM. There was no difference in the rate of significant out-degree CCEPs on MTG/STG between patients with or without SISCOM hyperperfusion on MTG/STG for early (*P* = 0.792), middle (*P* = 0.409) or late latency period (*P* = 0.817).

### Out-degree CCEPs to the closest RNS contacts

**Figure 6:**
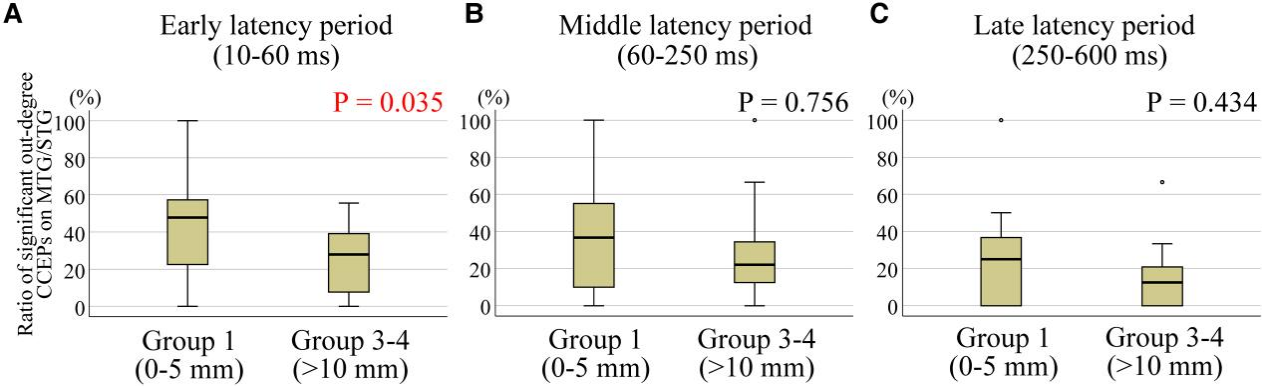
**Significant out-degree CCEPs and the correlation with the distance between stimulus site and the closest RNS contact.** Each data represents the ratio of significant out-degree CCEPs on MTG/STG, namely the number of stimulus pairs producing significant CCEPs divided by the number of all stimulus pairs. The stimulus sites with distances of 0–5 mm to the closest RNS contacts (G1: 0–5 mm) showed more significant out-degree early latency period than those with distances more than 10 mm to the closest RNS contacts (G3-4: >10 mm) (**A**) (*P* = 0.035). There was no difference in the significant out-degree CCEPs between G1 and G3-4 for middle (**B**) or late latency period (**C**) (*P* > 0.05, for both). A Wilcoxon Signed-rank test was used for all statistical tests. The numbers for each group for early, middle and late latency periods are 16.

The ratios of stimulus sites with significant out-degree during the early latency period over MTG/STG was compared to all stimulus sites based on the distance from SEEG CCEPs stimulus site to the closest RNS contact (grouped by G1 and G3-4). CCEPs in the G1 group (0–5 mm to the closest RNS contacts) showed more significant out-degree during the early latency period than those in G3-4 (>10 mm to the closest RNS contacts) (*P* = 0.035, a Wilcoxon Signed-rank test) (Fig. 6A). There was no difference in the significant out-degree CCEPs between the stimulus site in G1 and that in G3-4 for middle or late latency period (*P* > 0.05, for both) (Fig. 6B and C). We could only perform limited analysis of the seizure outcomes relating to out-degree CCEPs as described in Supplementary material as the regions of brain sampled and stimulated varied among the limited number of patients. A modest negative correlation was observed between the out-degree and the outcome in the late latency period, suggesting that better outcomes were associated with smaller out-degree CCEPs, when the electrodes in these two distance groups (G1 and G2) were stimulated (Supplementary Fig. 1). Also, we found a larger out-degree in patients with poor outcome (outcome scale 1), especially in the middle and late latency periods, for all three distance groups, excluding the late latency period in G2 (Supplementary Fig. 2).

## Discussion

### Correlation of CCEPs and RNS outcomes

In this study, we analysed CCEPs, during SEEG evaluations, in patients who subsequently underwent RNS therapy. CCEPs, a measure of evoked effective connectivity, enables an assessment of *in vivo* directional connectivity.^49^ Directional connectivity derived by CCEPs can be categorized as in-degree measures, representing the total number of significant responses evoked at a region of interest upon stimulation of all other sites, and out-degree CCEPs, representing the number of significant responses following stimulation of region of interest. We investigated the correlation between CCEPs and clinical characteristics including RNS contact locations and seizure outcomes from RNS therapy. The main findings of our study are as follows: (i) significant in-degree early latency CCEPs were seen at sites close to the eventual RNS electrode placement (Fig. 4A); (ii) a significant correlation was found between in-degree (in the early CCEPs latency period) and RNS seizure outcome, based on the distance from the eventual RNS contact location (Fig. 5A); and (iii) CCEPs stimulation sites within 0–5 mm of the eventual RNS electrodes showed more significant out-degree during the early latency period than those more than 10 mm away (Fig. 6A).

RNS electrode locations were placed based on expert analysis of SEEG IOZ localization. The CCEPs findings were not considered by the expert when determining the location of the RNS electrodes. The analysis of evoked functional connectivity in these patients, particularly the early latency period CCEPs, correlated well with clinically determined RNS electrode locations (both in-degree and out-degree) and degree of seizure reduction (only in-degree). This finding suggests that in addition to the standard clinical analyses of the IOZ, investigation of the effective connectivity by CCEPs could help guide the placement of electrodes for RNS therapy as an objective measure of the epileptogenic network.

### Mechanisms of RNS therapy

The observation of both acute as well as delayed therapeutic effects of RNS suggests that there are likely multiple mechanisms of actions.^14,63^ Examples of the acute effect of electrical stimulation include *in vitro* amplitude reduction of epileptiform activity,^64^ aborting after discharges^65^ and suppression of phase locked gamma-frequencies.^66^ A recent study has shown that acute stimulation related reduction in iEEG spectral power was associated with reductions in clinical seizure frequency.^67^ Such acute effects could be stimulus dependent and involve changes to neurotransmitters regulating the balance of excitation and inhibition.^68^ Electrical stimulation has also been shown to influence neuronal activity acutely distant from the site of stimulation.^66,69,70^ Progressive improvement in seizure control over years suggests that there are likely chronic effects related to electrical stimulation.^14-16^ Furthermore, one study found that modulation of the epileptic network over time appeared to correlate with changes noted in regions remote to the sites of stimulation.^71^ In a recent study focusing on the chronic network re-organization, patients with the greatest therapeutic benefit of RNS therapy showed progressive, frequency-dependent re-organization of interictal functional connectivity.^25^ In relation to the epileptic network, subregions of the cerebral cortex can be characterized based on the degree of incoming and outgoing connections.^48^ A key finding of our study is that RNS electrodes tend to be placed in regions of the cerebral cortex that could be described as major receiver nodes (receivers of influence) of large scale cortico-cortical influence. This conclusion is based on the correlations seen with early latency period in-degree CCEPs with eventual RNS electrode locations and seizure reduction. This finding is consistent with the observation that modulation of the epileptic network in regions distant to the site of stimulation may be important to the mechanisms by which responsive neurostimulation exerts its effects.^71^

Stimulus frequency may impact the therapeutic effect of electrical stimulation. Various stimulation frequencies have been studied for their potential neuromodulatory effects in epilepsy.^72-79^ Low-frequency SPES (∼1 Hz) also employed in CCEPs recordings has been shown to have inhibitory effects on ongoing epileptiform activity.^80,81^ High-frequency electrical stimulation has been shown to desynchronize neuronal activities.^64,70,82^ We studied the evoked effective connectivity using 1 Hz stimulation, while RNS therapy typically utilizes 100–200 Hz stimulation.^16^ Modelling changes in cortical excitability with repetitive stimulation (higher than 1 Hz) can also be used to understand evoked connectivity profiles, although RNS does not seek to study cortico-cortical connectivity using this high-frequency stimulation.^48^ Keller *et al*.^48^ showed that single-pulse evoked connectivity profiles provided a high degree of accuracy and discriminability of the cortical regions that exhibited changes in excitability to 10 Hz repetitive stimulation. In our study, we showed that single-pulse evoked connectivity profiles can also inform degree of seizure reduction in patients undergoing RNS (which typically utilizes higher frequency stimulation bursts). Greater in-degree CCEP, as a ratio distance, correlated with greater seizure reduction. A possible explanation of this finding is that these receiver node regions (higher in-degree) are more capable of being modulated by electrical stimulation than those regions of the brain with less in-degree properties.

### Assessing RNS electrode locations

Utilizing functional connectivity as a method for determining the appropriate settings of RNS therapy, including assessing potential sites of RNS electrodes, has previously not been established to the best of our knowledge. After the initial publications of CCEPs that evaluated various functional networks,^36,37^ CCEPs connectivity measures were used to study the epileptogenic network. SPES of the IOZ during seizure resulted in larger early CCEPs responses (within 50 ms after stimulation) than those during the interictal period, suggesting that the early latency period of CCEPs could be a marker of cortical excitability at the epileptic focus.^40^ A separate study comparing the early latency period of CCEPs at IOZ and non-IOZ regions showed that CCEPs at IOZ produced significantly higher amplitudes than non-IOZ regions.^41^ These accentuated CCEPs amplitudes, near IOZ, could reflect an increased cortical excitability associated with epileptogenicity. CCEPs connectivity was also studied in the propagation network of epileptic seizures. CCEPs evoked gamma band activity differed between early versus late ictal spread regions.^83^ CCEPs were also shown to have strong correlations between the evoked connectivity profiles to those areas with ictal hyperperfusion in SPECT.^43^ These studies strongly suggest that CCEPs could serve as markers of cortical excitability in epileptic networks. In our study, the MTG/STG regions were uniformly sampled by SEEG in all 12 patients. For this reason, we could study the out-degree of this region across patients. There was no difference in significant out-degree CCEPs with either epilepsy syndrome or ictal SPECT hyperperfusion. Although ictal SPECT hyperfusion is likely a measure of IOZ^18^ and seizure propagation,^84^ it is not a measure of directional connectivity. However, the early latency period out-degree CCEPs were significant in MTG/STG only when the RNS electrodes were placed within 5 mm of this region. This suggests that RNS electrode locations are targeted towards regions that serve as projection nodes within the brain.

## Summary

**Figure 7:**
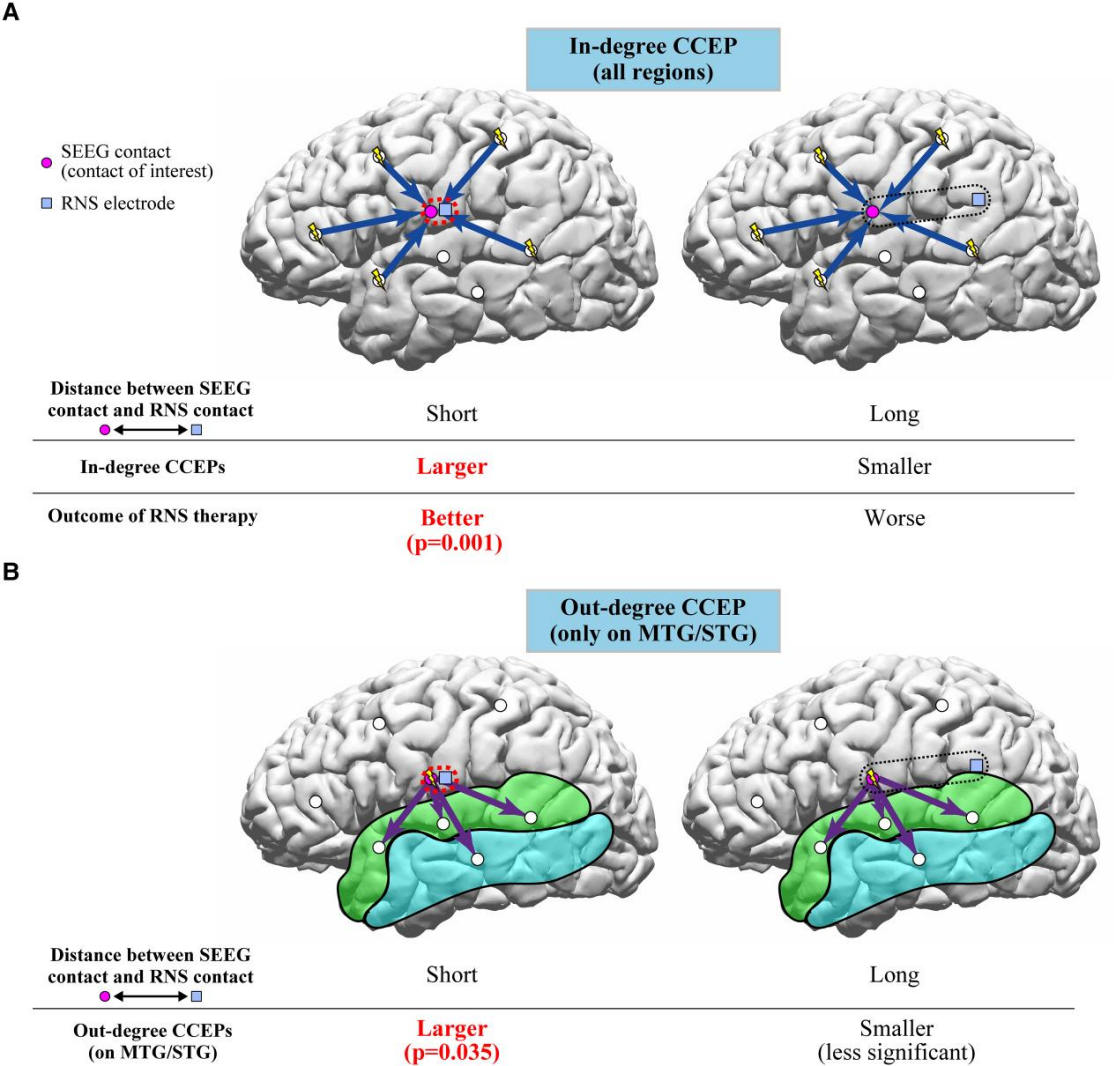
**Schema of interpretations of in-degree and out-degree CCEPs in relation to RNS therapy.** (**A**) The in-degree CCEPs recorded from contacts close to the RNS contact (G1) were larger than those recorded from the contacts relatively far from the RNS contact (G4) in six patients for early latency period. The ratio of amplitudes of early latency period in G1/G4 significantly correlated with the outcome of RNS therapy, suggesting that the recording sites involved in the network for the epileptic activities would be important for the RNS therapy. (**B**) The out-degree CCEPs by stimulating the sites with distances of 0–5 mm to the closest RNS contacts (G1) presented more significant early latency period than those with distances more than 10 mm (G3-4), implying that the CCEPs by stimulating the sites close to the RNS contacts reflect the cortical excitability at the site of stimulation and/or epileptic networks considering the pathological meanings of the early latency period of CCEPs.

In this study, we employed measures of in-degree and out-degree CCEPs and showed that both in-degree and out-degree responses correlate with the distances between the contacts of interest and the eventual RNS contact location. The in-degree CCEPs recorded from the contacts close to the eventual RNS contact tended to be larger compared with those recorded from the contacts further away. There were significant differences between G1 (<5 mm from the closest RNS contact) and G4 (>20 mm) in six out of 10 patients for CCEPs early latency period. The ratio of amplitudes of early latency period in G1/G4 showed a significant correlation with the outcome of RNS therapy. This is the first study showing correlations between RNS outcomes and effective connectivity measures using CCEPs (Fig. 7A) based on RNS electrode placement. We also demonstrated that locations planned by the clinical expert for RNS electrodes serve as receiver nodes within the brain. In a group analysis, the sites close to the eventual RNS contact location (G1 with distances of <5 mm to the closest RNS contacts) showed a larger number of significant out-degree CCEPs during the early latency period compared to those regions further away from the eventual RNS contact location (G3-4 with distances > 10 mm). Based on this finding, the location of RNS contacts could also reflect the cortical regions that serve as projection nodes to provide influence over wide regions of brain beyond the site of stimulation (Fig. 7B). These results of out-degree CCEPs do not show correlation with seizure outcome of RNS therapy but suggested that RNS electrodes might have been implanted in areas with higher out-degree to the MTG/STG. Additional analyses for out-degree CCEPs (Supplementary material), although limited, showed a larger out-degree in patients with poor outcome (outcome scale 1), especially in the middle and late latency periods, for all three distance groups, excluding the late latency period in G2. In our previous study,^46^ we found that focal cortical dysplasia (FCD) type I, generally associated with worse epilepsy surgery outcomes, exhibited more widespread and pronounced out-degree CCEPs. We also found that in patients with FCD type II, which has a better outcome after epilepsy surgery, had a more temporally and spatially restricted out-degree of CCEPs, suggesting a more focal epilepsy. For our current study, these findings imply that patients with poor outcomes from RNS therapy may show a pattern akin to that observed in FCD type I, characterized by a larger epileptic network. Thus, we can conjecture that those patients who responded positively to RNS likely had more focal epilepsy as well. Furthermore, the association between out-degree CCEPs and RNS outcomes was only seen in the late period of CCEPs. The pathophysiology of generation of the late CCEPs response is postulated to involve cortico-thalamo-cortical connections.^36^ It may be possible that the extent of involvement of subcortical networks in a given epileptic network might also affect RNS outcomes.

As described, the RNS electrodes were mainly implanted close to the IOZ, and thus other clinical measures of the IOZ are important in consideration of the RNS electrode location. However, this study suggests that functional connectivity exhibited by CCEPs could also help guide the placement of electrodes for RNS therapy. Since CCEPs can visualize the interictal effective connectivity in individual patients, their use to determine the targets for the RNS therapy appears reasonable when patients undergo SEEG evaluation before RNS therapy. While invasive evaluations may not be necessary in all the patients who will undergo implantation of RNS electrodes, presurgical invasive evaluation including IOZ and CCEPs analyses could be considered to better localize the RNS contact locations.

## Clinical implications and limitations

We acknowledge that this is a retrospective study with its inherent limitations. We showed that only in-degree CCEPs were correlated with favourable outcome of RNS therapy. The number of patients was limited and we could not analyse the seizure outcomes relating to out-degree CCEPs as the regions of brain sampled and stimulated varied among patients. This study was limited to patients who required an invasive evaluation for epilepsy. Many patients receiving RNS therapy do so without the need for an invasive evaluation therefore the results of this study would not be pertinent to that group of patients. The placement of SEEG electrodes was driven by the hypothesis of the epileptogenic network based on non-invasive data. A number of additional brain regions were sampled that were outside the ictal onset and propagation networks, however there is an inherent spatial limitation using SEEG. The limited number of regions of brain sampled by CCEPs led us to perform a group analysis for the out-degree CCEPs since an individual analysis could not be performed. We focused on the out-degree CCEPs on only the MTG and STG to standardize the responses among patients, who had variable probable epileptogenic zones and CCEPs stimulus sites. In the out-degree CCEPs analysis, evoked potentials from MTG and STG would be expected to be higher in amplitude if the stimulation site were near this region compared to those stimulation sites that were far away due to direct orthodromic propagation within cortico-cortical pathways. This could be an alternative explanation for this finding rather than it being representative of a significant projection node in the epileptogenic network. For the in-degree CCEPs analyses, we included the SEEG contacts in both grey and white matters, while most of the previous CCEPs studies during SEEG evaluations focused only on cortical responses. The clinical interpretation and significance of CCEPs recorded from white matter have not been clarified. One study, using subdural rather than SEEG electrodes, did show that subcortico-cortical evoked potentials could be elicited by stimulating the white matter.^85^ Epileptic activity can affect the integrity of white matter, which is one proposed mechanism involved in the emergence of cognitive and psychiatric co-morbidities.^86^ Therefore, we considered that it would be reasonable to include all SEEG contacts for the in-degree CCEPs analyses. Another limitation is that rather than using connectivity analysis for analysing distance between contacts, we used Euclidian distance because we do not have the connectivity analyses of these patients, such as a connectivity map or diffusion tensor image (DTI) for all the patients. The results we have presented in the current study show correlations between CCEPs and locations of RNS contacts likely reflecting the effective connectivity within the epileptogenic network. These measures were also correlated with favourable RNS treatment outcomes. The results of this study would indicate that CCEPs could provide additional insights in selecting appropriate stimulus sites for RNS therapy. Future investigations involving a larger number of patients and a greater number of CCEPs stimulation sites would be of interest and a prospective trial would be needed to clarify our findings in the future.

## Conclusions

Optimal electrode placement for RNS therapy is an important consideration in patients. In this study, evoked effective connectivity measures using CCEPs showed that the closer the distances of RNS contacts to the recording sites or stimulus sites of CCEPs, the larger the early latency in-degree CCEPs (receiver node) and significant early latency out-degree CCEPs (projection node). Electrophysiological characteristics of the epileptogenic network guiding the placement of RNS electrodes may very well play an important role in determining seizure outcomes with RNS therapy.

## Supplementary Material

fcae035_Supplementary_Data

## Data Availability

The de-identified data in this study is available from the corresponding author upon reasonable request and completion of a formal data sharing agreement.
