# Supplementary material for: Effective connectivity relates seizure outcome to electrode placement in responsive neurostimulation
Source: Brain Commun. 2024 Feb 22;6(1):fcae035. doi: 10.1093/braincomms/fcae035 (PMC10882982; doi:10.1093/braincomms/fcae035)
Supplement: fcae035_Supplementary_Data [file fcae035_supplementary_data.pdf]

Supplementary Material. An additional analysis for out-degree CCEPs.

Materials and Methods

We have performed a correlation analysis between normalized out-degree CCEPs and RNS outcomes although the out-degree analysis is limited since the number of stimulated contacts was not adequate for several patients. Due to the small sample size, group distances one and two (G1 and G2) were combined. Considering the relatively small patient sample size, the unavailability of outcome results for two patients, the categorical nature of the outcomes, and the limited number of stimulated electrodes, we analyzed individual bipolar files for out-degree CCEPs and used the Bootstrap method to generate more samples. For example, if there are 140 receiving channels, we can randomly select 40 and average them as a measure of out-degree, repeating this process 10 times for each stimulating channel.

The correlation of the out-degree CCEPs ratio with the outcome value of RNS therapy may not be particularly clear due to the limited outcome scales. To address this, we, in addition, compared the out-degree CCEP ratio between the group with surgical outcome 1 (comprising 3 patients) and the combined groups with outcomes 3 and 4 (also comprising 3 patients), across three latency periods: early, middle, and late. Using the

Bonferroni correction, the alpha level is 0.05/9 ≈ 0.006 in this test.

Results

Supplementary Figure 1 illustrates this relationship across three latency periods. This plot differs from its counterpart, Figure 5, in that it shows each stimulated bipolar contact rather than the average of all contacts in each patient. A modest negative correlation was observed between the out-degree and the outcome in the late latency period, after the Bonferroni correction ($\alpha = 0.05/3$), suggesting that better outcomes were associated with smaller out-degree CCEPs, when the electrodes in these two distance groups (G1 and G2) were stimulated.

Supplementary Figure 1. Correlation of the out-degree CCEPs ratio with the outcome value of RNS therapy

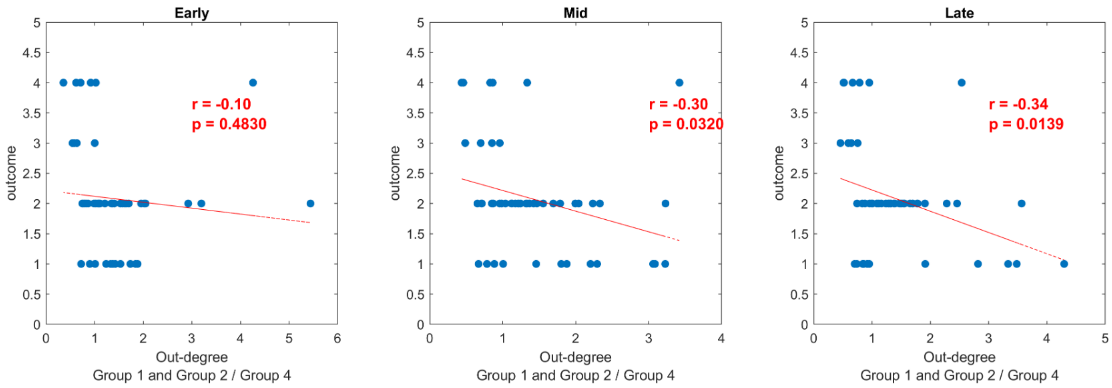

Supplementary Figure 2. Comparison of out-degree CCEP ratio between poor (scale 1) and better (scale 3 and 4) seizure outcome of RNS therapy

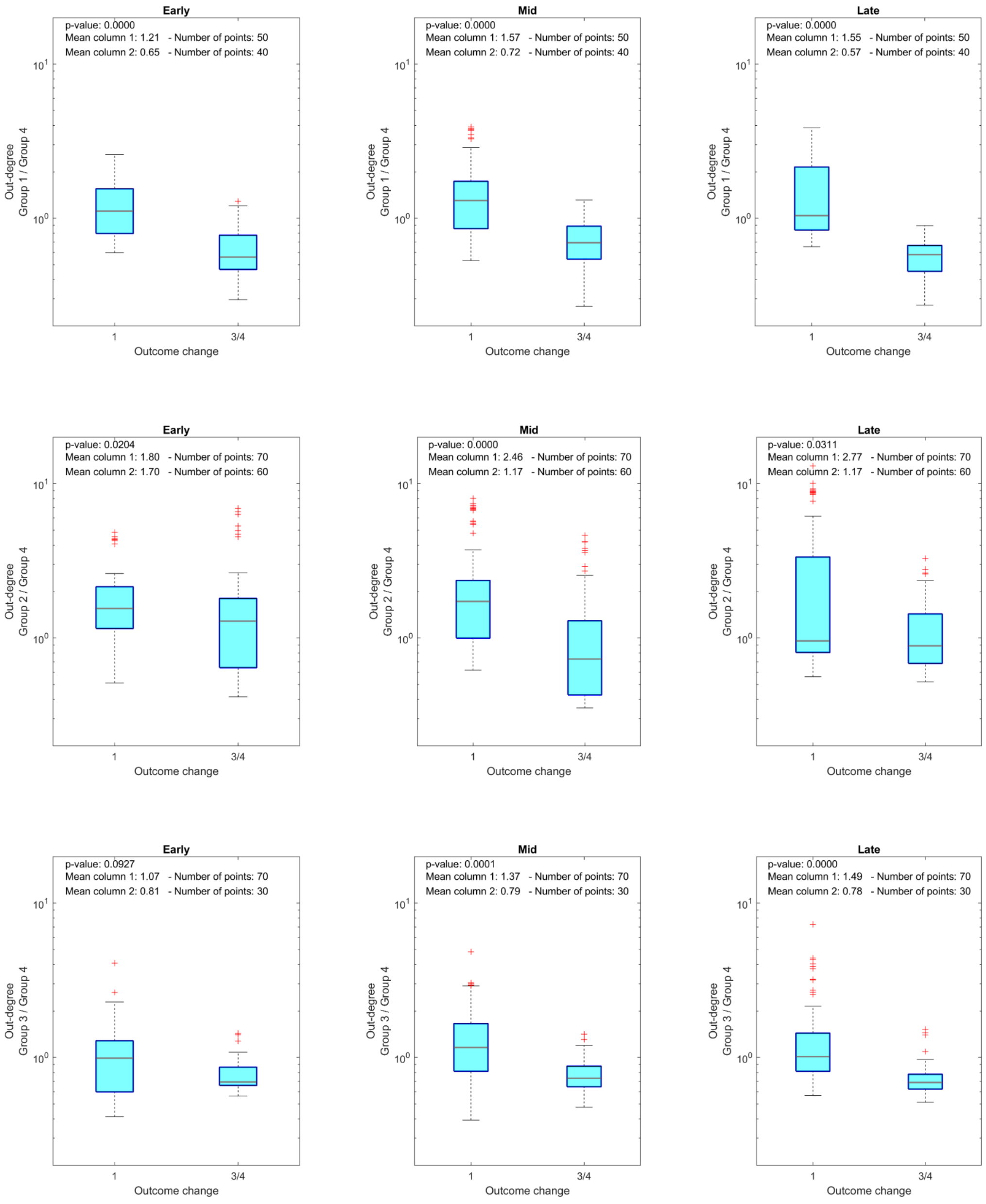

Supplementary Figure 2 features three plots, each based on electrode distance, namely G1/G4, G2/G4, and G3/G4. For improved visualization, the y-axis is presented in a logarithmic scale. These indicate a larger out-degree in patients with poor outcome

(outcome scale 1), especially in the middle and late latency periods, for all three distance groups, excluding the late latency period in G2.

Discussion

The observation of out-degree CCEP ratio between poor (scale 1) and better (scale 3 and 4) seizure outcome of RNS therapy can be supported by our previous study (Shahabi et al., Epilepsia. 2021), in which FCD type I - generally associated with worse outcomes - exhibiting a more widespread and pronounced response. Based on this, our current findings imply that patients with the poor outcomes from RNS therapy may show a pattern akin to that observed in FCD type I, characterized by a larger area of hyperexcitability. Conversely, we hypothesize that a more temporally and spatially restricted out-degree of CCEPs suggests more focal epilepsy (akin to FCD type II). Thus, we can conjecture those patients who responded positively to RNS likely had more focal epilepsy as well. Furthermore, the presence of strong late latency period in patients with the poor outcome might be related to the cortico-thalamo-cortical connectivity in language networks (Matsumoto et al., 2004).

Our results showed differences in out-degree CCEPs in three distance groups (up to 20 mm from the seizure onset zone), suggesting that the RNS electrodes might not have

targeted the precise location or that a larger area should have been targeted.

References:

Shahabi H, Taylor K, Hirfanoglu T, *et al*. Effective connectivity differs between focal cortical dysplasia types I and II. *Epilepsia*. 2021;62:2753-65.

Matsumoto R, Nair DR, LaPresto E, *et al*. Functional connectivity in the human language system: a cortico-cortical evoked potential study. *Brain*. 2004;127(10):2316-30.

Supplementary Table 1: Patient profile

Patient	Age at SEEG/CCEP	Age at RNS therapy	Gender	Epilepsy classification	Age of seizure onset	Risk factor	MRI	SISCOM in MTG/STG	Probable seizure focus	Lobar location of RNS electrodes (number of electrodes)	Ouctome change value
1	54	54	F	MTLE	6	-	-	-	Rt mesial temporal	Rt mesial temporal (1)	4
2	22	22	M	MTLE	13	head trauma	bilateral mesital temporal atrophy	-	Lt amygdala and Rt hemisphere	Lt mesial temporal (1) and Rt mesial temporal (1)	1
3	39	40	F	MTLE	18	head trauma	-	+	Rt mesial temporal	Rt mesial temporal (2)	n.a.
4	24	27	M	NTLE	10	perinatal event	Rt temporal-occipital FLAIR abnormality	-	Rt posterior basal temporal	Rt basal temporal (1) and Rt lateral temporal (3)	2
5	18	18	M	NTLE	14	-	-	+	Wide-spread especially Lt perisylvian	Lt basal temporal (1), Lt lateral temporal (2), and Lt lateral parietal (1)	2
6	26	26	M	NTLE	5	developmental delay	-	+	Bilateral posterior basal temporal	Lt basal temporal (1) and Rt basal temporal (1)	2
7	21	21	F	NTLE	7	-	-	+	Lt temporal-posterior perisylvian	Lt lateral temporal (2)	4
8	51	52	M	NTLE	late 20s	family history of epilepsy	-	-	Lt lateral temporal (STG)	Lt lateral temporal (3)	2
9	18	18	M	NTLE	16	head trauma	-	-	Lt and Rt lateral temporal (STG)-perisylvian	Lt lateral temporal (1) and Rt lateral temporal (1)	1
10	60	60	F	NTLE	34	-	-	+	Rt and Lt hemisphere	Lt insula (1), Rt posterior perisylvian (1), and Rt medial parietal (1)	n.a.
11	26	28	F	T-PLE	2	-	-	-	Lt posterior perisylvian-parietal	Lt lateral parietal (1) and Lt lateral temporal (2)	3
12	53	54	F	OLE	13	febrile seizures	-	not done	Rt occipital-posterior perisylvian	Rt occipital (2)	1

SEEG = stereoelectroencephalography; CCEP = cortico-cortical evoked potential; RNS = responsive neurostimulation; F = female; M = male; Rt = right; MTLE = mesial temporal lobe epilepsy; NTLE = neocortical temporal lobe epilepsy; PLE = parietal lobe epilepsy; OLE = occipital lobe epilepsy; SISCOM = subtraction ictal SPECT co-registered with MRI; MTG = middle temporal gyrus; STG = superior temporal gyrus; EZ = epileptogenic zone; Lt = left; n.a. = not available.

Supplementary Table 2: In-degree CCEPs ratios of G1/G4 and the outcome

	In-degree CCEP ratio (Group 1/Group 4)			Outcome change
	Early latency period (10-60 ms)	Middle latency period (60-250 ms)	Late latency period (250-600 ms)	
Patient 1	6.34	4.63	4.12	4
Patient 5	1.44	1.35	1.27	2
Patient 6	3.28	2.1	1.88	2
Patient 7	4.7	1.58	0.82	4
Patient 8	3.39	2.32	2.49	2
Patient 9	0.94	0.99	0.96	1
Patient 11	4.69	3.15	3.16	3
Patient 12	1.4	1.37	1.62	1
